# TIA-YOLOv5: An improved YOLOv5 network for real-time detection of crop and weed in the field

**DOI:** 10.3389/fpls.2022.1091655

**Published:** 2022-12-22

**Authors:** Aichen Wang, Tao Peng, Huadong Cao, Yifei Xu, Xinhua Wei, Bingbo Cui

**Affiliations:** ^1^ Key Laboratory of Modern Agricultural Equipment and Technology (Jiangsu University), Ministry of Education, Zhenjiang, China; ^2^ School of Software Engineering, Xi’an Jiaotong University, Xi’an, China; ^3^ Research and Development Department, Nanchang Huiyichen Technology Ltd., Nanchang, China

**Keywords:** weed detection, deep learning, object detection, YOLO, precision agriculture, site-specific weed management

## Abstract

**Introduction:**

Development of weed and crop detection algorithms provides theoretical support for weed control and becomes an effective tool for the site-specific weed management. For weed and crop object detection tasks in the field, there is often a large difference between the number of weed and crop, resulting in an unbalanced distribution of samples and further posing difficulties for the detection task. In addition, most developed models tend to miss the small weed objects, leading to unsatisfied detection results. To overcome these issues, we proposed a pixel-level synthesization data augmentation method and a TIA-YOLOv5 network for weed and crop detection in the complex field environment.

**Methods:**

The pixel-level synthesization data augmentation method generated synthetic images by pasting weed pixels into original images. In the TIA-YOLOv5, a transformer encoder block was added to the backbone to improve the sensitivity of the model to weeds, a channel feature fusion with involution (CFFI) strategy was proposed for channel feature fusion while reducing information loss, and adaptive spatial feature fusion (ASFF) was introduced for feature fusion of different scales in the prediction head.

**Results:**

Test results with a publicly available sugarbeet dataset showed that the proposed TIA-YOLOv5 network yielded an F1-scoreweed, APweed and mAP@0.5 of 70.0%, 80.8% and 90.0%, respectively, which was 11.8%, 11.3% and 5.9% higher than the baseline YOLOv5 model. And the detection speed reached 20.8 FPS.

**Discussion:**

In this paper, a fast and accurate workflow including a pixel-level synthesization data augmentation method and a TIA-YOLOv5 network was proposed for real-time weed and crop detection in the field. The proposed method improved the detection accuracy and speed, providing very promising detection results.

## 1 Introduction

During the process of crop growth, weeds appear randomly in the field and compete with crops for water, nutrients and sunlight, leading to a negative effect on crop yield and quality (Zhang, 2003; Lee et al., 2010). Research shows that weed competition may cause crop yield loss as high as 34% (McCarthy et al., 2010; Gao et al., 2018). Weed control has become one of the most important tasks in modern agricultural. Chemical and mechanical weeding campaigns are the two main means adopted at present. However, the overuse of herbicides by chemical weeding operations has resulted in serious environmental pollution problems. Mechanical weeding operations by tillage or cultivation of soil have been widely used for crops planted in rows, but they could hardly remove intra-row weed without assistance from a target detection module and may cause severe crop damage. In order to solve these problems, the concept of site-specific weed management (SSWM) was introduced, which could provide accurate target information for both the herbicide spraying system and mechanical weeding equipment (Lottes et al., 2018).

The key of SSWM is the rapid and accurate detection of target. For chemical weeding, the detection target is weeds, while for mechanical weeding, the detection target is crops for reducing crop damage rate. Therefore, rapid and accurate detection of both crops and weeds is very important for SSWM operations. At present, visible-near infrared (Vis-NIR) spectroscopy and machine vision are two main techniques used for crop and weed detection in the field. The Vis-NIR spectroscopy technique shows absolute advantage in detection speed, but it could hardly distinguish the weak spectral difference between crops and weeds in complicated outdoor environment. In addition, the detection region of Vis-NIR detector is limited, making this technique not suitable for crop and weed discrimination in the field (Wang et al., 2019). Compared with the Vis-NIR spectroscopy, machine vision could acquire information in a large area and provide accurate location information in the field of view. The image processing methods for machine vision can be grouped into conventional hand-crafted feature-based method and deep learning-based method. The conventional image processing method trains machine learning models based on color, texture, shape and other hand-crafted features. It has a simple model training process, but the model generalization ability and adaptability are low, hindering its practical applications under different circumstances. Moreover, the image preprocessing of the conventional method is cumbersome (Wang et al., 2022c). In recent years, with the rapid development of convolutional neural network (CNN) for image and video processing, deep learning-based image processing method has been widely studied and applied in the field of agricultural engineering (Wang et al., 2020a; Hasan et al., 2021). Deep learning-based object detector shows great advantages in target recognition, positioning and category determination. Jiang et al. (2020) established a grap convolutional network (GCN) map by extracting weed feature map and Euclidean distance through a CNN network, and enriched image features by using the GCN map. The recognition accuracy of GCN-ResNet-101 model on four weed datasets reached 97.80%, 99.37%, 98.93% and 96.51%, respectively, but the model convergence process was slow, and the model training was difficult. Furthermore, the network structure cannot be set too deep due to the limitation of the GCN network, otherwise it would cause the vanishing gradient problem. Kim and Park (2022) trained a multi-task semantic segmentation-convolutional neural network (MTS-CNN) model based on U-Net for the semantic segmentation of weeds and crops. Considering the large difference in the loss function between crops and weeds, they designed the cross-entropy loss and dice loss models between weeds, crops and both (weeds and crops) in the loss function stage to increase the stability of the network. The mean intersection over union (MIoU) trained on three public datasets was 91.61%, 83.72% and 82.60%, respectively. However, the model improvement was not based on the characteristics of specific objects (crops and weeds), resulting in poor generalization of the model. Peng et al. (2022) proposed an improved RetinaNet model to detect weeds among rice crops. The convolution structure was modified to reduce the loss of semantic information, the Efficient Retina Head (Lin et al., 2017) was designed in the head network to reduce memory consumption and inference time, and the regression loss function was designed by combining the Smooth loss (Girshick, 2015) and generalized intersection over union (GIoU) loss (Rezatofighi et al., 2019). Results showed that the average weed recognition accuracy of the model was 94.1%, which was 5.5% and 9.9% higher than the average recognition accuracy of the baseline network RetinaNet and YOLOv3, respectively. Although the model had some improvements in the prediction head, it did not make full use of the rich semantic information extracted from the backbone network, which cut the correlation between the feature maps. As a result, it was unfavorable for the practical application of the model.

Therefore, the overall objective of this work was to develop a fast and accurate model for weed and crop object detection in the field. Specifically, a pixel-level synthesization data augmentation method was proposed to deal with the problem of unbalanced sample distribution of weed and crop. An improved YOLOv5 network named the TIA-YOLOv5 was proposed, in which a transformer encoder block was added to the backbone to improve the sensitivity of the model to weeds, a channel feature fusion with involution (CFFI) strategy was proposed for channel feature fusion while reducing information loss, and adaptive spatial feature fusion (ASFF) was introduced for feature fusion of different scales in the prediction head. Lastly, the effectiveness of the proposed pixel-level synthesization data augmentation method and TIA-YOLOv5 network was tested with a publicly available sugarbeet dataset for sugarbeet and weed detection.

## 2 Materials and methods

### 2.1 Proposed method

YOLO series networks have been widely used in the field of agriculture for object detection (Wang et al., 2022b; Wang et al., 2022e). By now, the YOLO series models have developed into the seventh version, in which YOLOv5 is the most widely used for object detection. YOLOv5 is mainly composed of a (Cross-stage-Partial-connections) CSP-Darknet53 (Bochkovskiy et al., 2020) as the backbone, a path aggregation network (PANet) as the neck and a YOLO as the prediction head (Glenn, 2020). To improve the detection accuracy of crop and weed in the field, the YOLOv5s was selected as the baseline network in this work. The YOLOv5s is a light object detector with high performance, making it suitable for real-time object detection and easy to deploy on an edge computing platform in the field.

**Figure 1:**
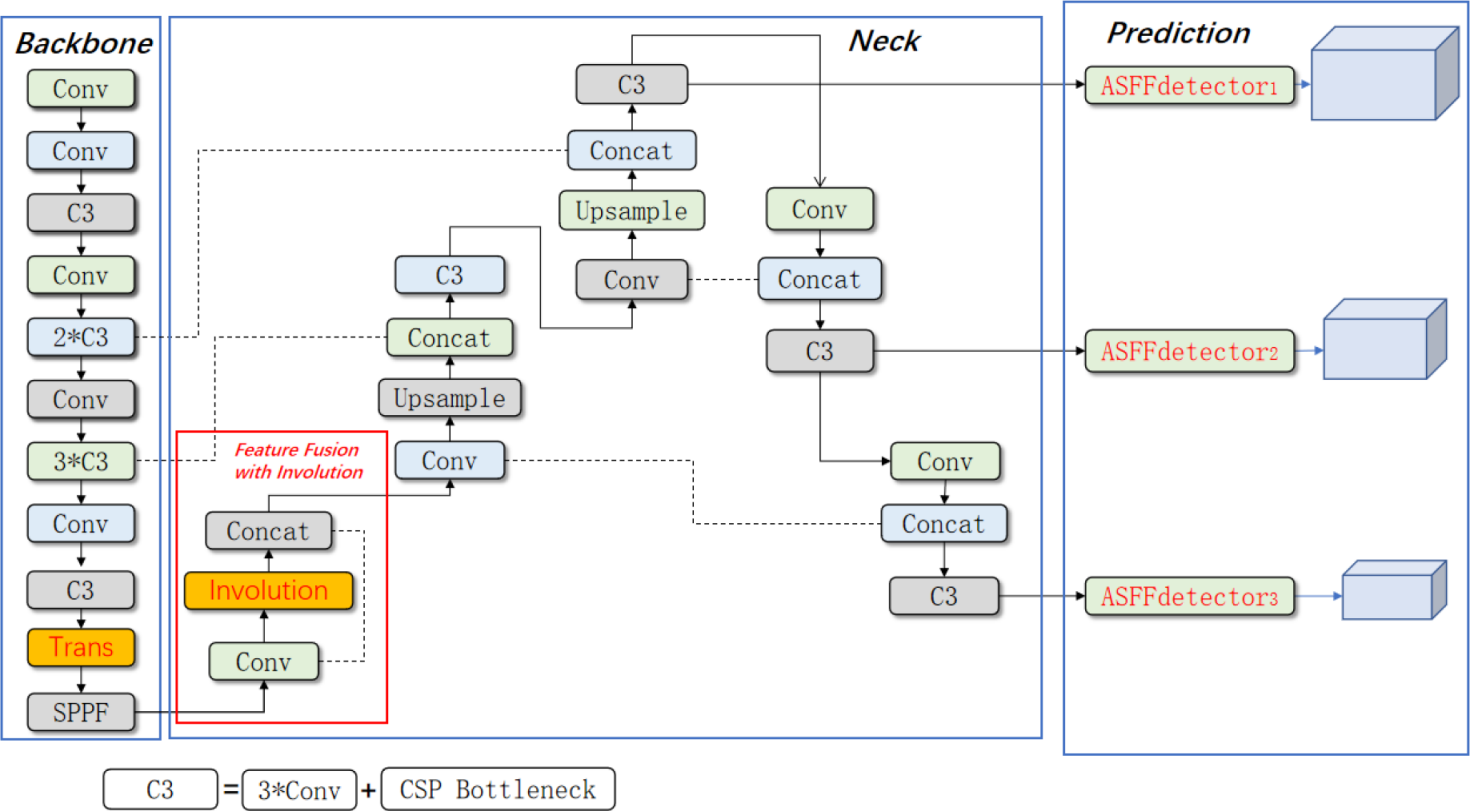
Architecture of the proposed TIA-YOLOv5 network. (* means multiply).

The architecture of the proposed TIA-YOLOv5 model is depicted in **Figure 1**. The model includes a backbone, a neck and a prediction head. To deal with the problems caused by occlusion, high density and sharp scale change in the current weed object detection scenario, the TIA-YOLOv5 network has three improvements compared with the baseline YOLOv5 model. Firstly, a transformer encoder block (Zhu et al., 2021) was added into the backbone to increase the feature extraction capability of the backbone network. Secondly, a channel feature fusion with involution (Li et al., 2021a) (CFFI) module was designed to reduce the loss of semantic information caused by convolution operations in the feature fusion stage and make full use of the rich information of the feature map at the end of the backbone. And thirdly, adaptively spatial feature fusion (Liu et al., 2019) (ASFF) was introduced to enhance the sensitivity of the prediction head to crops and weeds.

#### 2.1.1 Transformer encoder

**Figure 2:**
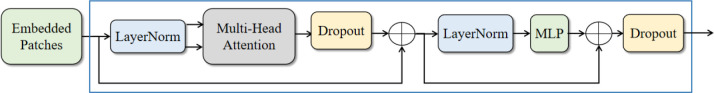
Structure of transformer encoder block (Zhu et al., 2021).

As the backbone of YOLOv5s, the CSP-Darknet53 is mainly composed of CSP bottleneck blocks, which is based on residual mechanism. The combination of transformer (Dosovitskiy et al., 2020) and CSP module is getting more attention by researchers recently due to its strong feature extraction capability. In the backbone, the transformer encoder block was deployed to replace the bottleneck of CSP-Darknet53. The transformer encoder block can get small target information better in the global information through self-attention mechanism, and has better detection performance in high-density crop images. The structure of the transformer encoder block is shown in **Figure 2**. It is mainly composed of a multi-head attention sub-layer and a multilayer perceptron (MLP) feed-forward neural network sub-layer with residual connections between them. In this work, the transformer encoder block was placed at the backend of the backbone, because the output of the backbone network is a low-resolution feature map, which will reduce the computational load of the transformer encoder block (Zhu et al., 2021).

#### 2.1.2 Channel feature fusion with involution (CFFI)

**Figure 3:**
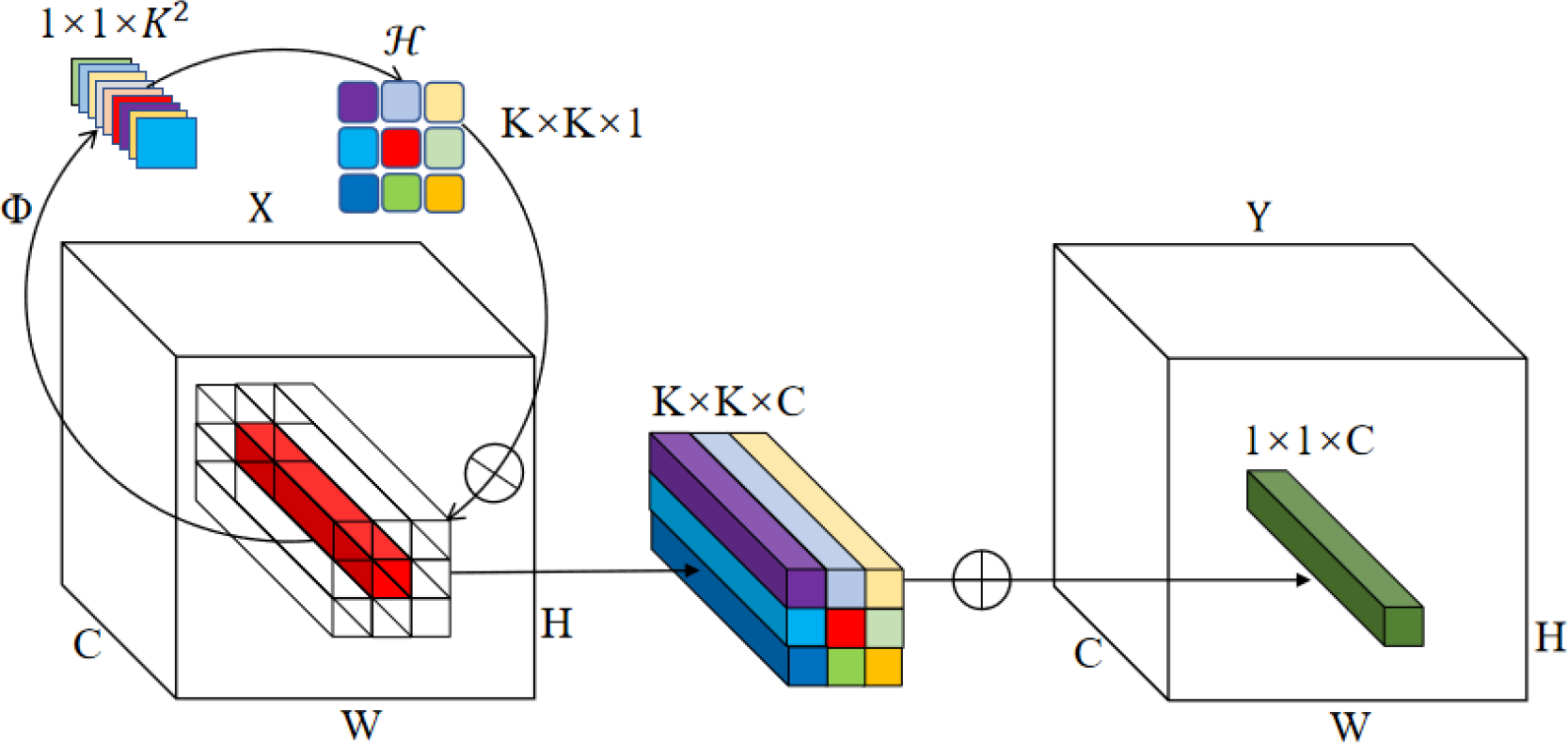
Schematic illustration of involution (Li et al., 2021a).

Path aggregation network (PANet) (Liu et al., 2018), which pools features from pyramid levels and fuses features of different scales, is widely used in object detection networks including YOLOv5. As the high-level feature map of the backbone, feature {C5} contains rich semantic information. To integrate with the mapping of the feature {C4} in the backbone, 1 × 1 convolution layers were adopted to reduce the channel number of feature {C5}, through which the calculation efficiency was significantly improved. However, the reduction of channel number would inevitably result in serious information loss (Luo et al., 2022). To reduce this information loss, the CFFI was added between the backbone and feature pyramid, which enabled the semantic information to be fully utilized. Based on this concept, in order to take full advantage of the rich features in the high-level channels of the backbone and improve the performance of the PANet, involution operation (Li et al., 2021a) was adopted. Involution is characterized by light weight, high efficiency and flexibility, and has achieved good results in various visual tasks. Different from convolution operation, involution has channel invariance and spatial specificity, enabling it to overcome the difficulty of modeling long-range interactions (Li et al., 2021a). The formula of the involution kernel is *ℋ*_*i*,*j*_∈*ℝ* ^*K*×*K*×1^ , where *ℋ*_*i*,*j*_ generates according to the function Φ at a pixel (*i, j*), *K* is the kernel size of involution. The structure of involution is shown in **Figure 3**. In involution, the size of the input feature map directly determines the size of involution kernel, therefore the size of the kernel and the input feature map automatically aligns in the spatial dimension. The advantage of this is that the involution kernel can adaptively allocate the weights over different positions (Li et al., 2021a).

**Figure 4:**
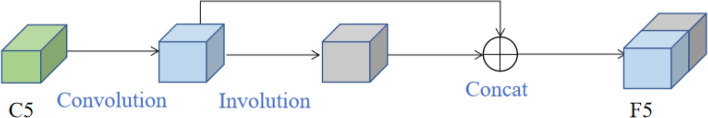
Channel feature fusion with involution.

It was observed that the channel number of the feature map {C5} can maximize the performance of involution. Therefore, the CFFI was introduced to directly reuse the rich semantical information of the feature {C5} (Luo et al., 2022). Firstly, a 1 × 1 convolution with channel number of 768 was used to reduce the channel number of the feature {C5}. Then an involution with kernel size of 1 × 1 and channel number of 768 was used to aggregate spatial information. Finally, a concat operation was conducted to connect the resulted feature maps of convolution and involution, as shown in **Figure 4**. Through these operations of CFFI, the channel number of feature maps was reduced, as well as the loss of semantic information caused by direct convolution. Therefore, the CFFI functioned as a bridge between the backbone and neck, which enhanced the representation ability of the feature pyramid.

#### 2.1.3 Multiscale feature fusion

**Figure 5:**
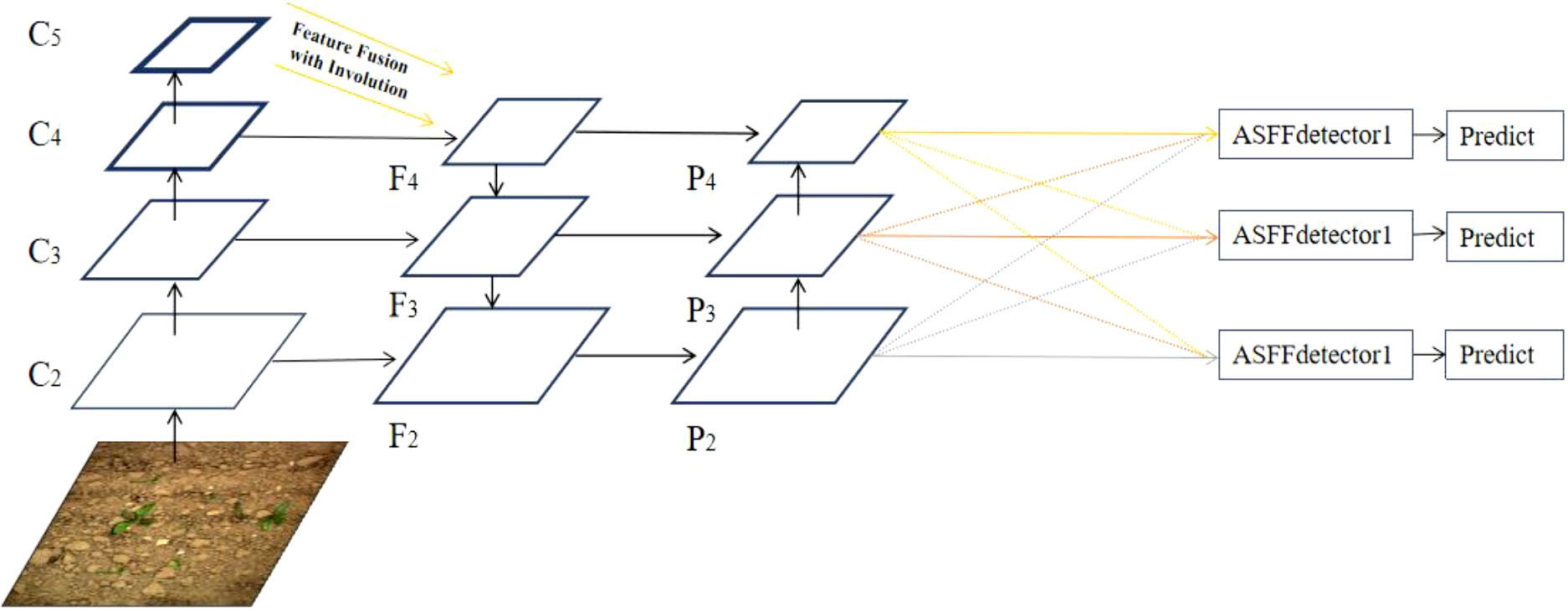
Schematic diagram of adaptively spatial feature fusion (ASFF).

YOLOv5 is a single-stage detector. The main problem of YOLOv5 is the inconsistency between feature maps of different scales (Liu et al., 2019). For weed detection task, there are large scale differences between crops and weeds, resulting in inaccurate positioning and identification of objects. In order to make full use of the multi-layer features output from the neck, the adaptively spatial feature fusion (ASFF) (Liu et al., 2019) was introduced. Unlike the bi-directional cross-scale connections and weighted feature fusion (BIFPN) and learning scalable feature pyramid architecture (NAS-FPN) that use cascaded multi-level feature fusion, features of different scales were rescaled and adaptively fused in ASFF to filter out the inconsistency during training. The structure of the ASFF is shown in **Figure 5**.

There are three resolutions of feature maps in YOLOv5, ***P*_2_**, ***P*_3_**, and ***P*_4_**. For feature map of *P*_*i*_(*i*∈{2, 3, 4}) , the resolution of other *P*_*n*_(*n*≠*i*) feature maps was adjusted so that all feature maps have the same size. The ASFF modifies the upsampling and downsampling strategies for the three feature maps. For up-sampling, the 1 × 1 convolution was used to reduce the channel number of feature maps, then interpolation was conducted to improve the resolution of feature maps. Two downsampling strategies were used; For the feature map of 1/2 scale, the 3 × 3 convolution with a stride of 2 was used to modify the channel size and resolution simultaneously, and for the feature map of 1/4 scale, the maximum pooling with a stride of 2 was added before the convolution layers with a stride of 2. The last step of ASFF was to adaptively learn the spatial weights of feature map fusion under multiple scales for adaptive fusion (Liu et al., 2019). The formula of ASFF is as Eq. (1).


(1) $$y_{ij}^{l}=\alpha_{ij}^{l}\cdot x_{ij}^{1\to l}+\beta_{ij}^{l}\cdot x_{ij}^{2\to l}+\gamma_{ij}^{l}\cdot x_{ij}^{3\to l}$$

Where $y_{ij}^{l}$ is the (i,j)-th vetor of the output feature map *y* ^*l*^ between channels, and $x_{ij}^{n\to l}$ represents the feature vector at the position (*i, j*) on the feature map adjusted from the *n* level to the level. $\alpha_{ij}^{l}$ , $\beta_{ij}^{l}$ , $\gamma_{ij}^{l}$ , are the spatial weights corresponding to the three levels of feature maps, which were obtained by network adaptive learning. $\alpha_{ij}^{l}$ , $\beta_{ij}^{l}$ and $\gamma_{ij}^{l}$ are subjected to the Eq. (2).


$$\alpha_{ij}^{l}+\beta_{ij}^{l}+\gamma_{ij}^{l}=1$$

(2) $$\alpha_{ij}^{l},\;\beta_{ij}^{l},\;\gamma_{ij}^{l}\in \;\left[0,1\right]$$

$$\alpha_{ij}^{l}=\frac{e^{\lambda_{a_{ij}}^{l}}}{e^{\lambda_{a_{ij}}^{l}}+e^{\lambda_{\beta_{ij}}^{l}}+e^{\lambda_{\gamma_{ij}}^{l}}}$$

By adopting this strategy, adaptive fusion was carried out on each feature scale, which greatly reduced the instability of the model caused by the scale changes between crops and weeds.

### 2.2 Dataset preparation

#### 2.2.1 Image dataset

In this work, a publicly available sugarbeet image dataset (Chebrolu et al., 2017) was used. The images were captured with a readily available agricultural robotic platform, BoniRob, on a sugar beet farm near Bonn in Germany in spring 2016. All images are in RGB format and the resolution is 1296 pixel × 966 pixel. The dataset includes images of sugar beet at seedling and growth stages as well as weeds in the field (Chebrolu et al., 2017). In this study, a total of 4500 images were selected as our raw dataset. LabelImg (https://github.com/tzutalin/labelImg) was used to label crop and weeds in the images and provide corresponding label files for model training.

#### 2.2.2 Image preprocessing

**Figure 6:**
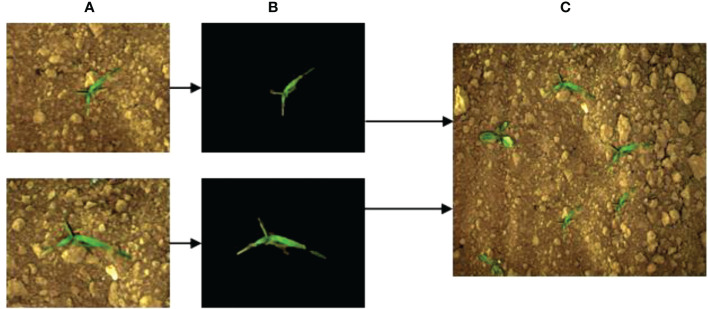
Image augmentation process for increasing the number of weed objects. **(A)** Sub-image containing only weeds and background, **(B)** Extracted weed pixels, **(C)** Synthetic new images containing more weed objects.

The balance of samples determines the robustness of the trained models. During the process of labeling the sample data, it was found that the sample number of weeds and sugarbeet in the public image dataset was seriously unbalanced, with the ratio of sugarbeet to weeds was about 17:3. To increase the number of weed samples, a pixel-level synthesization data augmentation method was designed. Six kinds of weeds with different sizes and shapes were extracted from original images, then these weed pixels were inserted randomly into images to synthesize new images containing more weed objects. The data augmentation process is shown in **Figure 6**. Sub-images (**Figure 6A**) that only contained weeds and background were cropped from the original images. The background and weeds in the sub-image were then segmented using the region growth method (Angelina et al., 2012). The extracted weed pixels are shown in **Figure 6B**. These extracted weed pixels were merged into original images randomly to obtain new images (**Figure 6C**) containing more weed objects. After data augmentation, the number of total images reached 5536, and the sample ratio of sugarbeet to weeds was 17:9.

The enhanced dataset containing 4100 original images and 1436 synthetic images was divided into a training set, a validation set and a test set with a ratio of 8:1:1. Since the pixel-level synthesization data enhancement method is a process of data expansion based on the original images, to avoid the repetition of weeds and crops in the training set and test set, the obtained synthetic images and corresponding original images that were used for date augmentation were only used for model training. As a result, the resulting training, validation and test set contained 4395, 500 and 641 images, respectively.

### 2.3 Experiment design

The model training and testing tasks in this work were conducted on a PC equipped with a 11th Gen Intel i9-11900K CPU and a NVIDIA GTX3080Ti GPU. Pytorch 1.10.2 framework was used to build the networks. For parameter settings, the pretrained weights of YOLOv5s on the COCO dataset was loaded as initial weights. The input image size was 1296 × 966, the training epochs was set to 100, and the batch size was set to 8. To verify the effect of the data augmentation method on the proposed TIA-YOLOv5, conventional data augmentation method including random translation, rotation and scaling was also used for comparison. Six groups of ablation experiments were conducted to analyze the effects of each module of the TIA-YOLOv5 network. Finally, the TIA-YOLOv5 were also compared with state-of-the-art object detection models including SSD (Liu et al., 2016), Faster RCNN (Girshick, 2015), YOLOv3 (Redmon and Farhadi, 2018), YOLOv4 and YOLOv7 (Wang et al., 2022a) networks.

### 2.4 Evaluation metrics

Precision, recall, average precision (AP), mean average recognition accuracy (mAP@0.5 and mAP@0.5:0.95), F_1_-score and processing speed in terms of frame per second (FPS) were used to evaluate the model performance. The mAP is the average of the AP calculated for all the classes, where N is the number of categories. The mAP@0.5 means that the mAP calculated at intersection over union (IoU) threshold of 0.5. The mAP@0.5:0.95 means the average mAP over IoU thresholds from 0.5 to 0.95 with an interval of 0.05. The formulas for precision, recall, F_1_-score, AP and mAP are as follows.


(3) $$Precision=\frac{True\;Positive}{True\;Positive+False\;Positive}\times 100\%$$

(4) $$Recall=\frac{True\;Positive}{rue\;Positive+False\;Negative}\times 100\%$$

(5) $$F1-score=2\times \frac{Percision\times Recall}{Percision+Recall}\times 100\%$$

(6) $$AP=\int_{0}^{1}Percision\left(Recall\right)d\left(Recall\right)\times 100\%$$

(7) $$mAP=\frac{\sum_{1}^{N}\int_{0}^{1}Percision\left(Recall\right)d\left(Recall\right)}{N}\times 100\%$$

## 3 Results and discussion

### 3.1 Performance of dataset augmentation

**Figure 7:**
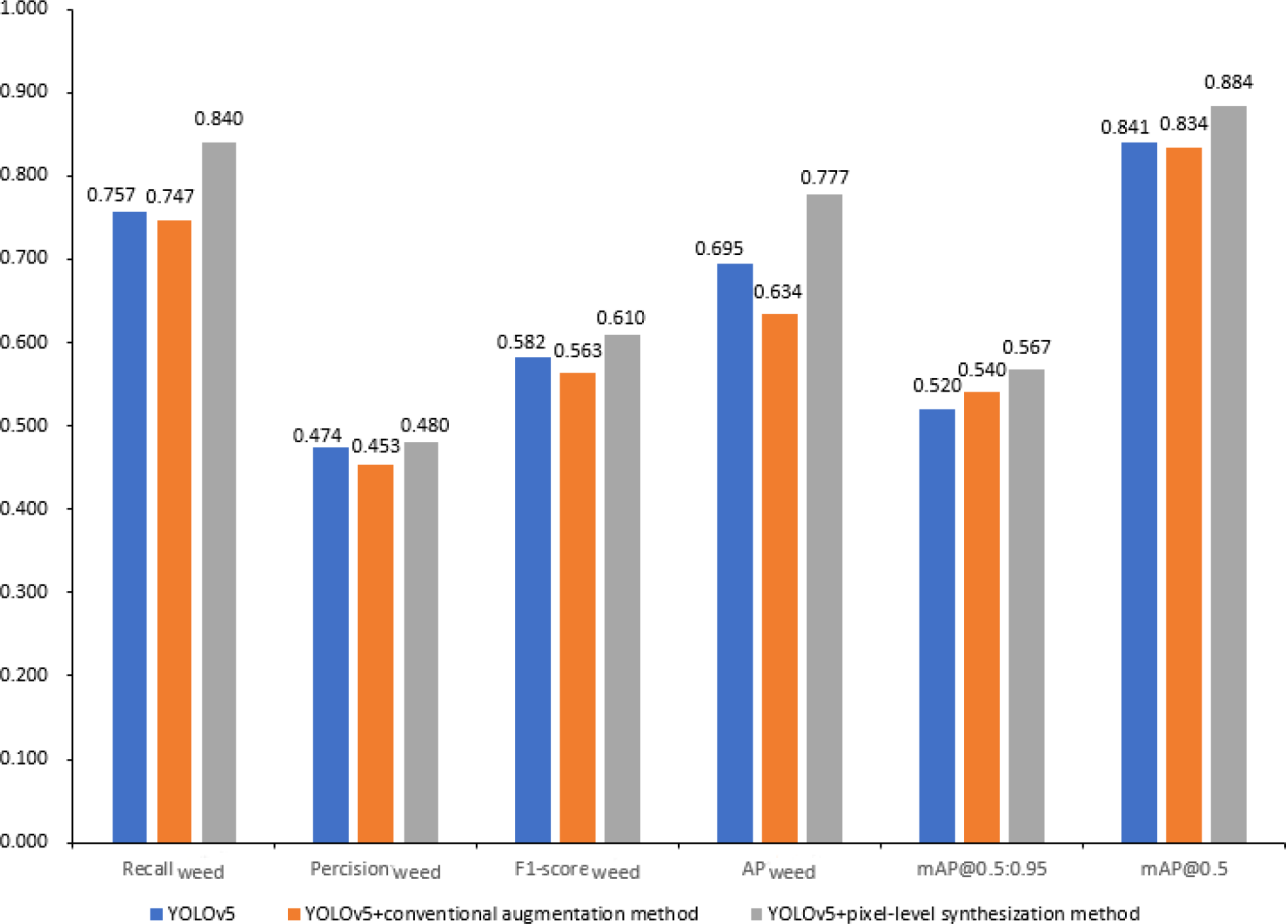
Performance of data augmentation methods.

To evaluate the performance of the proposed pixel-level synthesization dataset augmentation method, commonly used conventional dataset augmentation methods including random translation, rotation and scaling were used for comparison. The number of samples in the training, validation and test set for each method was set the same. The results are shown in **Figure 7**. Because the precision, recall, F_1_-score, and AP values for detecting sugarbeet were all above 90% for all models, they were not listed and discussed as performance indicators. The YOLOv5 model without data augmentation performed slightly better than the ‘YOLOv5+conventional augmentation method’ model. This is because the ‘YOLOv5+conventional augmentation method’ replaced 1436 original images with corresponding processed images by the conventional data augmentation method to keep the same number of samples in the training set. The proposed pixel-level synthesization method were superior to the conventional method in terms of all the six indicators. The conventional data augmentation method could enhance the dataset to some extent, however, the lack of weed samples that results in the unbalanced data distribution is the key problem in this work, which cannot be solved by the conventional augmentation method. The pixel-level synthesization method was designed to increase the number of weed samples in the dataset, further to alleviate the unbalanced phenomenon of data distribution. From **Figure 7** it can be observed that the F_1_-score_weed_, AP_weed_ and mAP@0.5 were improved to 61.0%, 77.7% and 88.4%, with 4.7%, 14.3% and 5.0% absolute increase compared with the conventional data augmentation method, respectively, validating the effect of the pixel-level synthesization method.

### 3.2 Ablation study

Ablation study was conducted to evaluate the effect of different modules of the proposed TIA-YOLOv5 network. The results of ablation study are shown in **Table 1**, from which it can be seen that the Recall_weed_, F_1_-score_weed_, AP_weed_, mAP@0.5 and mAP@0.5:0.95 of the ‘YOLOv5+Transformer’ model increased by 11.3%, 3.6%, 8.3%, 4.4% and 5.9%, respectively, compared with the baseline YOLOv5 network. These improvements indicate that the transformer encoder block could improve the sensing ability of the YOLOv5 network for weeds, making it easier to capture the weed objects from the complicated context information. In addition, the calculation cost of the transformer encoder block was less than that of the original bottleneck module of the YOLOv5 network, which improved the FPS of the model. The combination of the pixel-level synthesization augmentation method and transformer encoder block reduced the additional computer resource consumption caused by the increase of samples. Although adding the CFFI module alone cannot improve the model performance on identifying crops and weeds significantly, the Recall_weed_, F_1_-score_weed_, AP_weed_ and mAP@0.5 of the ‘YOLOv5+Transformer+CFFI’ model reached 86.0%, 62.5%, 79.8% and 89.4%, with an absolute increase of 10.3%, 4.3%, 10.3% and 5.3% compared with the baseline network, when combining the transformer encoder block with the CFFI module. This is because the CFFI module could make full use of the rich context information captured by the transformer encoder block, realizing the reuse of the information extracted from the backbone network and further providing rich front-end information for feature fusion layers in the neck (Zhu et al., 2021). The ASFF module could further optimize the network by applying multi-scale feature fusion in the prediction head. In this work, the feature maps of were fused to reduce the instability of the model caused by the scale difference of feature maps (Liu et al., 2019). With the combination of the transformer encoder block, CFFI and ASFF modules, the proposed TIA-YOLOv5 provided the best performance among the compared networks, with the Recall_weed_, F1-score_weed_, AP_weed_ and mAP@0.5 of 90.0%, 70.0%, 80.8% and 90.0%, respectively. Comparing the TIA-YOLOv5 with the baseline network, the improvement of mAP values is not very evident. This is because the mAP is determined by the detection accuracy of both crop and weed, and the AP_crop_ is already 99.2% for the baseline network. With respect to the F1-score_weed_, AP_weed_, the absolute improvement by the TIA-YOLOv5 is 11.8% and 11.3% compared with the baseline network, which is a promising result for weed object detection in the field. In general, the TIA-YOLOv5 network could provide higher weed detection accuracy and remain comparable processing speed relative to the YOLOv5 network.

**Table 1:** Ablation study on the effect of different modules of the proposed TIA-YOLOv5 network.

Model	Recall_weed_	Precision_weed_	F_1_-score_weed_	AP_weed_	mAP@0.5	mAP@0.5:0.95	FPS
YOLOv5	0.757	0.474	0.582	0.695	0.841	0.520	21.5
YOLOv5+Transformer	0.870	0.481	0.619	0.778	0.885	0.579	21.7
YOLOv5+CFFI	0.840	0.480	0.610	0.779	0.885	0.581	19.1
YOLOv5+Transformer+CFFI	0.860	0.492	0.625	0.798	0.894	0.586	20.2
YOLOv5+Transformer+CFFI+ASFF	0.900	0.573	0.700	0.808	0.900	0.580	20.8

### 3.3 Comparison with other object detection networks

The proposed TIA-YOLOv5 was compared with the SSD, Faster RCNN, YOLOv3, YOLOv4 and YOLOv7 networks in terms of Recall_weed_, Precision_weed_, F1-score_weed_, mAP@0.5 and FPS to verify its effectiveness. The results are shown in **Table 2**. The Faster RCNN is a two-stage object detector that consists of a region of interest (ROI) generation step and a feature extraction step. It has been applied in agriculture for object detection tasks (Li et al., 2021b; Zhao et al., 2022). For the weed and crop detection task in this work, the Faster RCNN yielded an F1-score_weed_ of 60.7%, an mAP of 86.6% and an FPS of 5.8. The detection accuracy is competitive, however, the detection speed is too slow, which should be caused by the separated regression and classification networks of the Faster RCNN (Wang and Liu, 2021), making this model hardly to be used in the field for real-time weed detection. The SSD is a one-stage object detector, and is significantly faster than the Faster RCNN in FPS. However, the SSD did not perform well for small target detection, with the F1-score_weed_ 14% and the mAP 8.6% lower than the TIA-YOLOv5, because it tended to use the low-level feature maps for small target detection. The YOLOv3 (Redmon and Farhadi, 2018) is the most classical network in the YOLO series, and the baseline YOLOv5 in this work was developed from YOLOv3. The YOLOv3 with squeeze-and-excitation networks (SE-YOLOv3) (Hu et al., 2018) uses feature pyramid networks (FPN) to fuse feature maps of different levels. The FPN has a relatively simple architecture and does not need much computing resource, enabling the SE-YOLOv3 has an absolute superiority in detection speed while maintaining acceptable detection accuracy (Wang et al., 2020b). With the appearance of PANet, the combination of the FPN and PANet has significantly improved the model performance on the cross-scale fusion (Wang et al., 2022d). The YOLOv4 network also uses this strategy. Compared with the SE-YOLOv3 and YOLOv4 models, although our proposed TIA-YOLOv5 network performed slightly inferior in FPS, the mAP@0.5 increased by 6.4% and 3.7%, respectively. The newly emerged YOLOv7 network adopts the extended efficient layer aggregation networks (E-ELAN), yielding an F1-score_weed_ of 60.4% and an mAP of 87.2%. It was superior to all the compared networks expect the TIA-YOLOv5. With respect to processing speed, when deployed on a Jetson NX (NVIDIA, US) computing platform, the TIA-YOLOv5 model could yield an FPS of about 70 after optimization by the TensorRT SDK (https://github.com/NVIDIA/TensorRT) provided by NVIDIA, which can meet the requirement of real-time application in the field. Overall, our proposed TIA-YOLOv5 provided the best performance for sugarbeet and weed detection among the five compared models.

**Table 2:** Performance comparison of five networks for sugarbeet and weed detection.

Network	Recall_weed_	Precision_weed_	F_1_-score_weed_	mAP@0.5	FPS
Faster RCNN	0.810	0.486	0.607	0.866	5.8
SSD	0.740	0.451	0.560	0.814	12.4
SE-YOLOv3	0.800	0.481	0.600	0.836	24.3
YOLOv4	0.835	0.472	0.603	0.863	21.2
YOLOv7	0.780	0.493	0.604	0.872	19.2
TIA-YOLOv5	0.900	0.573	0.700	0.900	20.8

**Figure 8:**
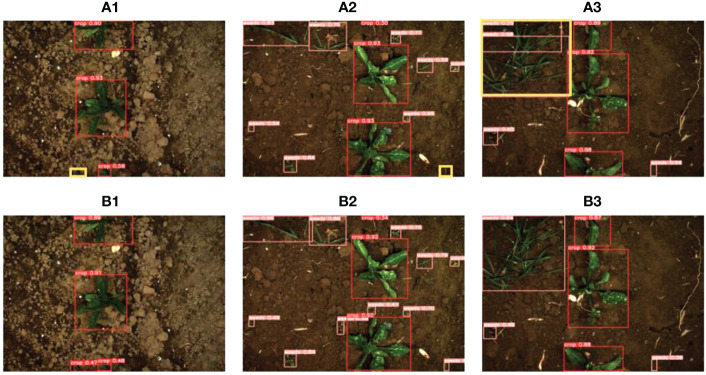
Visualization of YOLO5 (Figures a) and TIA-YOLOv5 (Figures b) for detecting sugarbeet and weed.

Several images were selected from the test set for visualizing the detection performance of the TIA-YOLOv5 network, as shown in **Figure 8**. The yellow boxes in **Figures 8** a1 and a2 are the weeds that were missed by the baseline YOLOv5 network but detected by our TIA-YOLOv5. The yellow box in **Figure 8** a3 is the region where weed was detected repeatedly by the YOLOv5 while the TIA-YOLOv5 detected them correctly. From this visualization comparison between the YOLOv5 and TIA-YOLOv5, it can be observed that the detection of small object is a challenging task and the YOLOv5 tends to miss these small objects. By adding the transformer encoder block, CFFI and ASFF, the TIA-YOLOv5 has stronger ability for sensing small objects and could effectively avoid problems such as missed, false and repeated detection, making this model suitable for real-time detection of weeds and crops in the field.

## 4 Conclusion

In this work, a fast and accurate workflow was proposed for weed and crop object detection in the field. Specifically, a pixel-level synthesization data augmentation method that generated synthetic images by pasting weed pixels into original images was proposed to deal with the problem of unbalanced data distribution of weed and crop. An improved YOLOv5 network named the TIA-YOLOv5 was developed for weed and crop objection detection. In the backbone of the TIA-YOLOv5, a transformer encoder block was used to improve the sensitivity to small weed objects. In the neck, the CFFI was proposed for channel feature fusion and reducing information loss. In the prediction head, the ASFF was introduced for feature fusion of different scales. Test results with a publicly available sugarbeet dataset showed that the proposed TIA-YOLOv5 network yielded an F_1_-score_weed_, AP_weed_ and mAP@0.5 of 70.0% 80.8% and 90.0%, respectively, which was 11.8%, 11.3% and 5.9% higher than the baseline YOLOv5 model. And the detection speed reached 20.8 FPS. When deployed on a Jetson NX computing platform, the TIA-YOLOv5 model could yield an FPS of about 70 after optimization by the TensorRT SDK, which is very promising for real-time weed and crop detection in the field. Future work will be focused on developing an SSWM system incorporating the trained weed detection model.

## Data availability statement

The data presented in this study are available on request from the corresponding author.

## Author contributions

Conceptualization, AW and TP; methodology, AW, TP, and YX; software: TP and YX; validation, AW and TP; formal analysis, AW, TP, and HC; investigation, TP and HC; resources, AW, XW, and BC; data curation, TP and HC; writing—original draft preparation, TP and HC; writing—review and editing, AW; visualization, TP; supervision, AW and YX; project administration, AW and XW; funding acquisition, AW. All authors have read and agreed to the published version of the manuscript.
